# HPV-Stratified Causal Digital Twins for Personalized Survival Benefit Estimation in Head and Neck Cancer

**DOI:** 10.64898/2026.09.30.26363910

**Published:** 2026-10-02

**Authors:** Keyur D. Shah, Chih-Wei Chang, Jun Zhou, Jie Ding, Anees Dhabaan, Hania A. Al-Hallaq, James Bates, William Stokes, David S. Yu, Xiaofeng Yang

## Abstract

**Purpose:**

Digital-twin frameworks can potentially offer a pathway toward individualized treatment but remain largely untested in radiation oncology. We present a causal digital-twin model for head-and-neck (H&N) cancer that estimates patient-specific survival benefit from chemoradiotherapy (CRT) versus radiotherapy (RT) alone, stratified by HPV status.

**Methods:**

Using the public RADCURE cohort (n = 3,346), we developed a causal digital-twin framework in which patients were divided into HPV-positive and HPV-negative groups and assigned to training (RT start year ≤ 2007) and test sets (RT start year > 2007). Bayesian-network structure learning identified each cohort’s Markov Blanket around the 2-year survival endpoint (SVy2), yielding the minimal causally relevant feature set. A causal-forest model (double machine learning, DML framework) estimated the individualized treatment effect *τ* = *P* ( *Svy2* | *CRT,X* ) − *P* ( *Svy2* | *RT,X* ), with denoting the model-estimated effect. Overlap restrictions were applied to mitigate extrapolation. Predictive discrimination (AUC, C-index), average treatment effects (ATE), individualized effects (ITE), and decision-curve analyses were computed.

**Results:**

For HPV-negative disease, internal evaluation demonstrated strong discriminative performance (test AUC = 0.72; C-index = 0.81, p < 0.01). The estimated test-set ATE favored CRT with a modest but positive improvement in SVy2 (mean ). Patterns in were clinically coherent, including increased benefit in node-positive and good-performance patients and reduced benefit in high-burden or poor-performance presentations. Decision-curve analysis showed that a -guided selective-CRT strategy provided higher net benefit than uniform treatment policies. HPV-positive disease exhibited substantially greater but less clinically structured treatment-effect heterogeneity, accompanied by reduced overlap and greater uncertainty.

**Conclusion:**

This study demonstrates a proof-of-concept causal digital-twin framework for interpretable, patient-specific survival simulation in H&N cancer. The model captures clinically plausible treatment-effect heterogeneity and establishes a foundation for multi-cohort validation and future integration into individualized treatment decision support.

## Full Text Availability

The license terms selected by the author(s) for this preprint version do not permit archiving in PMC. The full text is available from the preprint server.

